# A micro-bioimpedance meter for monitoring insulin bioavailability in personalized diabetes therapy

**DOI:** 10.1038/s41598-020-70376-5

**Published:** 2020-08-12

**Authors:** Pasquale Arpaia, Umberto Cesaro, Mirco Frosolone, Nicola Moccaldi, Maurizio Taglialatela

**Affiliations:** 1 CIRMIS - Interdepartmental Center for Research in Management and Innovation in Healthcare, University of Naples Federico II, Naples, Italy; 2 Department of Electrical Engineering and Information Technology, University of Naples Federico II, Naples, Italy; 3 Department of Public Health and Preventive Medicine, University of Naples Federico II, Naples, Italy; 4 Department of Neuroscience and Reproductive and Odontostomatological Sciences, University of Naples Federico II, Naples, Italy

**Keywords:** Drug delivery, Biomedical engineering

## Abstract

An on-chip transducer, for monitoring noninvasively the insulin bio-availability in real time after administration in clinical diabetology, is proposed. The bioavailability is assessed as insulin decrease in situ after administration by means of local impedance measurement. Inter-and-intra individual reproducibility is enhanced by a personalized model, specific for the subject, identified and validated during each insulin administration. Such a real-time noninvasive bioavailability measurement allows to increase the accuracy of insulin bolus administration, by attenuating drawbacks of glycemic swings significantly. In the first part of this paper, the concept, the architecture, and the operation of the transducer, as well as details about its prototype, are illustrated. Then, the metrological characterization and validation are reported in laboratory, in vitro on eggplants, ex vivo on pig abdominal non-perfused muscle, and in vivo on a human subject, using injection as a reference subcutaneous delivery of insulin. Results of significant intra-individual reproducibility in vitro and ex vivo point out noteworthy scenarios for assessing insulin bioavailability in clinical diabetology.

## Introduction

For people living with diabetes (PWD), a full control of blood glucose level is a challenge of wide clinical, social, and economic importance^1^.

The Artificial Pancreas (AP) represents a promising response to this need^2^. AP, known as closed-loop control of blood glucose in diabetes, combines a glucose sensor, a control system, and an insulin infusion device (Insulin Pump). Glucose control is aimed at measuring the patient’s need to adjust insulin dosage, or even to shut down insulin delivery in case of hypoglycemia (e.g. caused by exercise, miscalculated insulin bolus). The loop is closed in case of basal insulin administration. In case of meals, even the most recent automated systems cannot react to the quick variations of glucose due to food ingestion. Both for commercially available and do-it-yourself artificial pancreas systems meal-time boluses need to be manually delivered by the user—this is why the systems are still called “hybrid-closed loop systems”^3,4^. A generic bolus calculation algorithm needs personalized control inputs as: Total Daily Dose of insulin (TDD), carbintake, basal/bolus ratio, basal segments, carbohydrate-to-insulin ratio (CIR), target range, insulin sensitivity factor (ISF), and insulin duration of action. In particular, the levels of ISF and insulin duration are fixed during the initial calibration of the algorithm. However, they can be subject to relevant variations depending on the kinetics of the insulin absorption.

Intra- and inter-individual variability in insulin absorption are well-known drawbacks for therapy in PWD. Several factors contribute to such a variability, including insulin type, bolus size, injection site, and the presence of skin alterations, such as lipo-hypertrophic nodules^5^. A more accurate administration of insulin bolus can be guaranteed by a real-time monitoring of the amount of drug actually absorbed, namely bioavailable. However, the knowledge of the insulin quantity actually bioavailable does not represent an exhaustive answer to: (1) glutoxicity problems (insulin effect is lower in case of excessive hyperglycemia), and (2) the miscalculation of bolus, because the carbohydrate content is not estimated correctly (significant problem in diabetes, especially when eating out in restaurants).

To date, main methods for assessing insulin bioavailability are enzyme-linked immunosorbent assay^6^, radioimmunoassay^7^, and high-performance liquid chromatography^8^. Unfortunately, these methods are invasive or have high latency^9^.

In a recent work, the feasibility of a method to measure the amount of drug administered transdermally by impedance spectroscopy was demonstrated^10^. Then, the Drug Under Skin Meter (DUSM), a general-purpose bioimpedance meter able to determine the amount of drug transmitted transdermally, was proposed^11^. The DUSM was proved to be able to assess the amount of drug (ml) delivered in a test tissue, by measuring the impedance spectrum before and after drug administration. However, twofold critical drawbacks of non-reproducibility need to be taken into consideration, at intra-individual (different body areas, skin conditions, psychological conditions, and so on), as well as inter-individual (sex, age, ethnicity, and so on) level.

In this paper, an on-chip bio-impedance transducer, for real-time noninvasive monitoring of insulin bio-availability after administration in clinical diabetology, is presented. Drug administration in known doses by insulin pump allows to identify a specific personalized impedance-model for each tissue single condition (insulin appearance model). The insulin model is employed during absorption measurement for assessing insulin disappearance, and thus bio-availability. This allows to improve significantly inter- and intra-individual reproducibility. In Sections I and II, the concept and the physical design, as well as the realization of hardware, firmware, and software, are illustrated respectively. Then, in Sections III the metrological. characterization is reported, laboratory impedance comparison, in-vitro on eggplants ex-vivo on pig abdominal non-perfused muscle and in vivo on a human subject.

## Design

In this section, the "Basic ideas", the "Architecture", and the "Operation" of the insulin meter are illustrated.

### Basic ideas

According to the well-known concept of bioimpedance^12,13^, a tissue is considered as an electrolytic solution containing a certain number of cells. In a solution, the amount of solute is assessed by measuring impedance. Similarly, in a biological tissue, the measurement of impedance variation is used as a measure of the amount of insulin. This concept underlies the following ideas^10^:

1. The insulin bioavailability over time is assessed indirectly from the measurement of its time dependent disappearance from the administration volume of biological tissue.
2. This insulin variation is assessed noninvasively by electric impedance variation. The leakage of a given amount of insulin (ml) produces a corresponding variation in the measured equivalent impedance in the administration volume. (Owing to its low linearity and monotonicity, the phase is not considered, and only the impedance magnitude is used in the measurement).
3. The relationship between the variation of impedance and the insulin decrease is linear^14^.
4. At each administration, a linear model for the individual subject in each his/her condition is identified (personalized medicine). This increases significantly inter- and intra-individual reproducibility of bioavailability measurements.
5. The specific personalized linear model is characterized metrologically in order to verify the reproducibility, resolution, sensitivity in assessing the insulin amount.
6. The administered drug is assumed as a single element, formed by units of insulin, saline solution and excipients. In^14^, the insulin variation per ml in solution was proved to vary according to the impedance magnitude, in an electrolytic cell and in eggplants.

### Architecture

**Figure 1:**
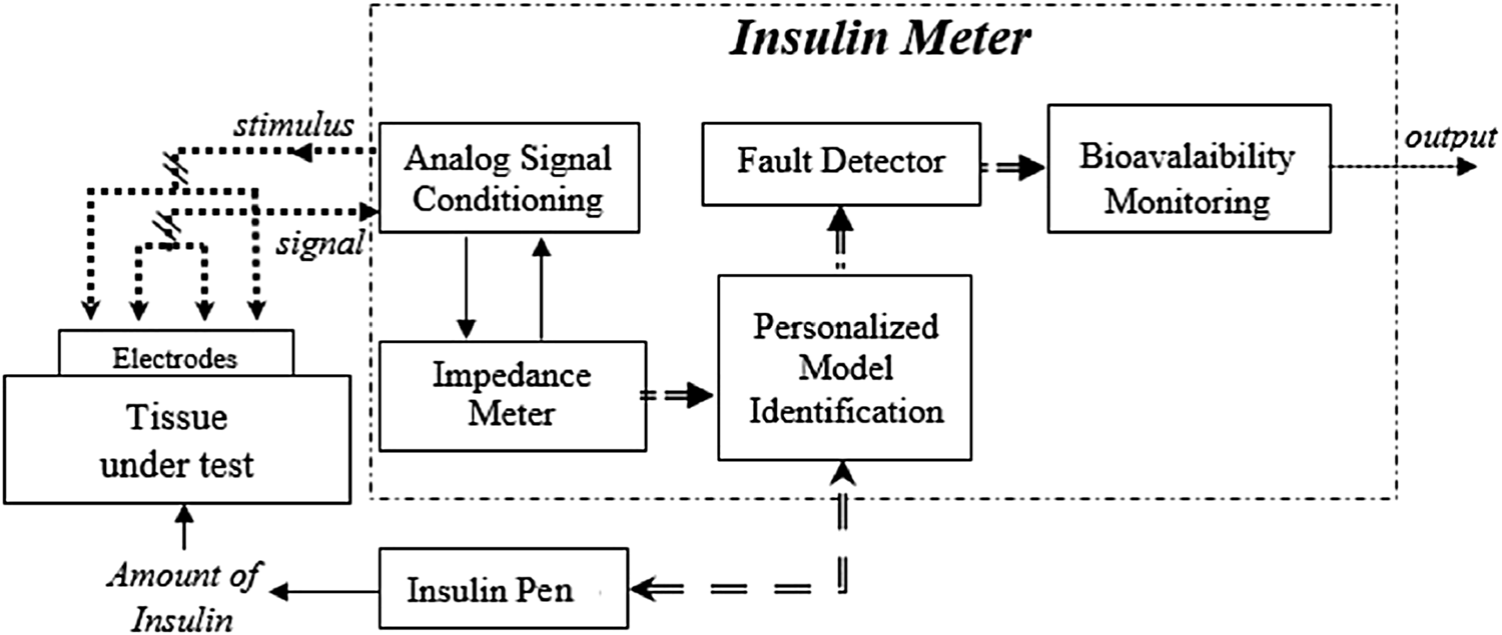
Architecture of the insulin meter.

The architecture of the proposed insulin meter is shown in Fig. 1.

Four *Electrodes* are connected to the skin of the subject where the insulin was injected (*Tissue Under Test*). The *Impedance Meter* sends a sinusoidal current stimulus with given amplitude and frequency through the amperometric electrodes, and takes the corresponding voltage drop through the voltage meters, through the *Analog Signal Conditioning*. The insulin is injected at known step by an *Insulin Pump*, and step by step the transducer measures the corresponding impedance. On this basis, the insulin meter carries out a *Personalized Model Identification*. Then, in the *Fault Detector*, the personalized model parameters identified experimentally during the injection are checked to fall within the uncertainty limits assessed by off-line metrological characterization. In positive case, the transducer is able to measure the amount of insulin absorbed by the tissue, through an assessment of the impedance variation. Finally, the bio-available insulin is assessed by difference, by monitoring the reduction of the initial amount over the time (*Bioavalaibility Monitoring*).

### Operation

The insulin meter measurement includes the following steps:

- *Electrode application* The four-electrode configuration reduces the uncertainty impact of the skin-electrode interface. FIAB PG500 electrodes (Vicchio, Florence, Italy) were used to facilitate the anchoring of the electrodes to the surface, minimize the movement artifacts, and reduce the difference in impenetrability. These electrodes have a bio-compatible conductive gel, consisting of demineralized deionized water, a complexing agent (EDTA), a thickening agent (Carbomer), a neutralizing agent (sodium hydroxide), and NaCl. A custom 3D-printed positioner was exploited to make the measurement setup stable.
- *Reproducibility enhancement* the impedance average over each measurement cycle is used to attenuate the impact of the intrinsic variability of biological structures, as well as to reduce the uncertainty of a single measurement. As a matter of fact, body cells change their electrical behavior according to thickness and hydration of the stratum corneum. Then, each average value is normalized to the pre-injection mean value, in order to make the personalized model specific to the single subject in that particular physiological condition.
- *Model construction* at a frequency of 1 kHz, the impedance variation is measured at each step of consecutive insulin injections. The model relates impedance variation to insulin amount; this calibration model is identified by the injection process. In this way, a linear relationship is obtained that identifies the variation of impedance in relation to the quantity of drug injected^10^. This model is identified specifically for each subject, in the particular physiological condition of each injection, in order to enhance inter-and- intra-individual reproducibility.
- *Fault detection* verification that the linear model prediction falls within tolerance intervals defined during the preliminary metrological characterization of the insulin meter.
- *Bioavailability montoring* the impedance changes is mesured over time, to determine the relative change in insulin using the previously constructed linear model.

## Realization

The transducer was prototyped by using all off-the-shelf components. Basic components of the Analog Device were used: a motherboard EVAL-ADUCM350-REV A, and a daughterboard BIO3Z Vers.A with attached FIAB PG500 electrodes for measurement.

### Hardware

**Figure 2:**
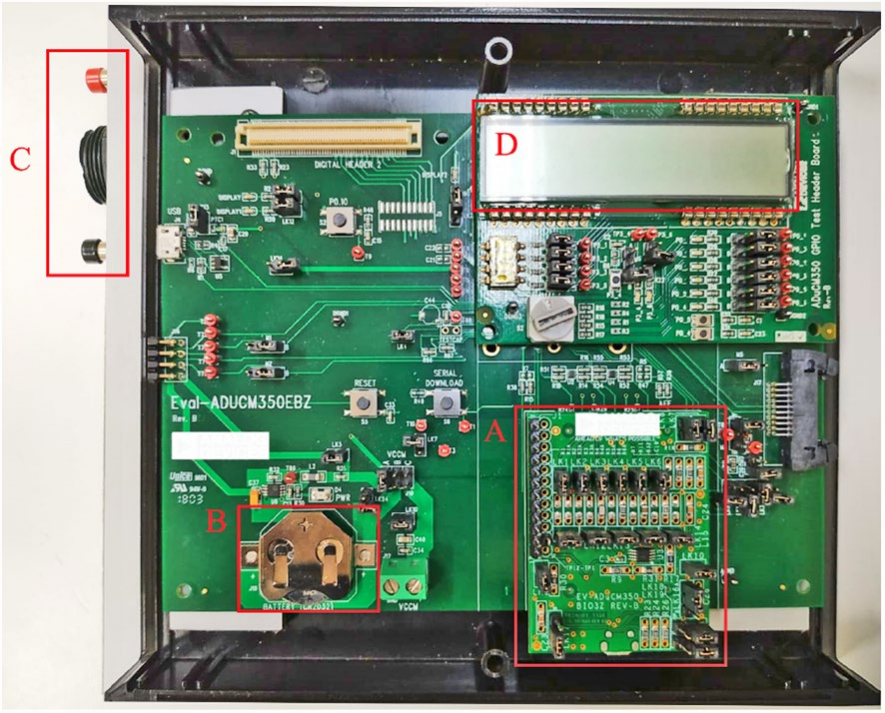
Prototype board of the insulin meter: motherboard ADuCM350 Vers.A with (**A**) daughterboard BIO3Z for 4-wire bioimpedance measurement, (**B**) battery, (**C**) ON and Reset buttons, and (**D**) display.

The AduCM350 Vers.A is a motherboard, with a processor ARM Cortex-M3 at 16 MHz, equipped with an analog front-end. The motherboard has a 16-bit DAC to impose the sinusoidal input signal and a 16-bit to 160 kS/s ADC converter, capable of converting the detected current signal into a voltage signal. The motherboard is also able to perform a DFT on 2048 points calculating both the real part and the phase of the complex impedance^12,13^. The transducer (Fig. 2) has a slot for the daughterboard of signal conditioning. At this aim, 4-wire measurements are carried out by adopting the daughterboard “4-Wire Bio Impedance Configuration 4W-BIO3Z” Fig. 2(A).

The transducer has three power supply options: USB cable, battery, and 3.3 V via external transformer and wall socket. For Holter monitor use, i.e to measure impedance variation over long term, the battery-powered solution is adopted Fig.2(B). Furthermore, the user can verify the measurement process by means of an 8-digit ADuCM350-GPIO-REV-B LCD of the Analog Device and a selector. The IEC 60601-1 standard for the safety of electromedical equipment with the patient’s active part specifies the limits of leakage currents under normal and faulty conditions. The standard indicates twofold ranges limits depending on whether the stimulus frequency $f_e$ lower is or higher than 1 kHz. In the first case, the maximum RMS current allowed by the standard is 10 $\upmu$A; conversely:

$$\begin{aligned} f <= 1 {\text {kHz}},&\qquad I_{MAX}=10\upmu {\text{A}} \\ f >1 {\text{kHz}},&\qquad I_{MAX}=\frac{f_e}{1000 {\text{Hz}}} * 10 \upmu {\text{A}} \end{aligned}$$

By an additional resistance, the transducer remain always below the thresholds imposed by the standard.

### Firmware

Analog Devices provides libraries for basic operations using the BIO3Z board, i.e. carry out 4-wires impedance measurements. On this basis, a suitable firmware to operate the transducer was developed. The through-put was increased by initializing the analog front-end only at the measurement start. Afterwards all the measurements are saved in the internal flash memory, postponing data transmission only at the end of the measurement. In this way, acquisition time was reduced down to 34%. Two easily-accessible ON and Reset buttons are provided to force start or stop of a measurement by hand during debug Fig. 2(C). A set of ten different programs was created to make the measurement easier. The display allows the user to view the current program and the measurement number in place Fig. 2(D). Up to 7500 16-bytes measurements can be saved on the transducer memory spaces of 120 kByte. The PC access via COM port and UART management protocol allows to download the measurements.

## Metrological characterization

The insulin meter was characterized metrologically (1) *in laboratory*, through comparison with reference impedance, (2) in vitro, on eggplants, (3) ex vivo, on pigs, and (4) preliminary in vivo, on a human subject, through insulin infiltration.

### Laboratory tests

#### Experimental setup

In laboratory tests, the transducer performance was compared with the 4263B LCR Meter from Agilent Technologies as a reference. As equivalent circuit, a parallel between a capacitor [0.5 nF] and a variable resistor [42–2500 $\Omega$] was realized through a 1433-M Decade Resistor and a Standard Capacitor Type 509-F both of General Radio.

#### Calibration

32 measurements were carried out, for each resistor value, at the frequencies of [100, 120, 10,000, 10,000, 20,000] Hz, with a sine wave amplitude of 100 mV, at a temperature of ${23}^{\circ }\hbox {C}$ and humidity of 50%. Average and standard deviation of the impedance module of the RC loop were assessed, at varying the resistor value linearly within [42, 2500] $\Omega$.

#### Results

**Figure 3:**
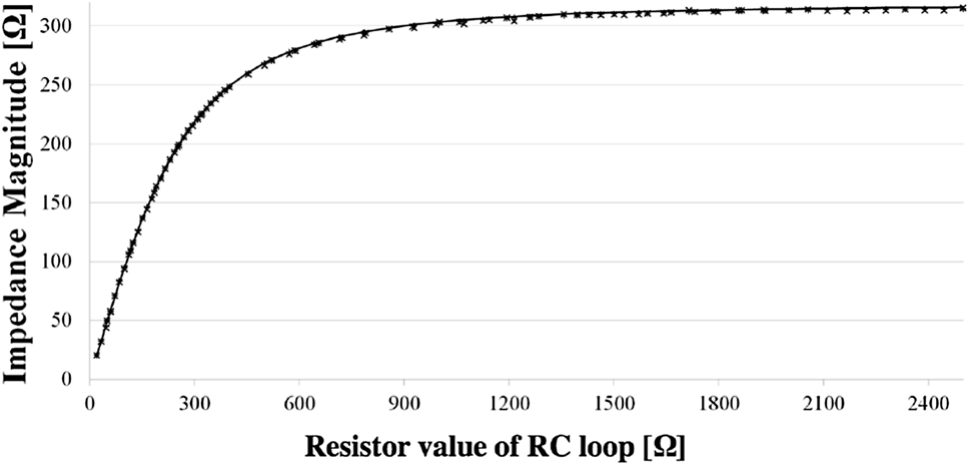
Impedance magnitude at 1 kHz at varying the resistor value of the RC loop: theoretical trend (continuous line), insulin meter ($\times$), and reference LCR Meter (+).

Figure 3 shows the 1 kHz impedance variation measured by the Agilent and the InsulinMeter in comparison with the nominal values at 1 kHz for an RC loop. The instruments present a compatible trend with the nominal one.

**Figure 4:**
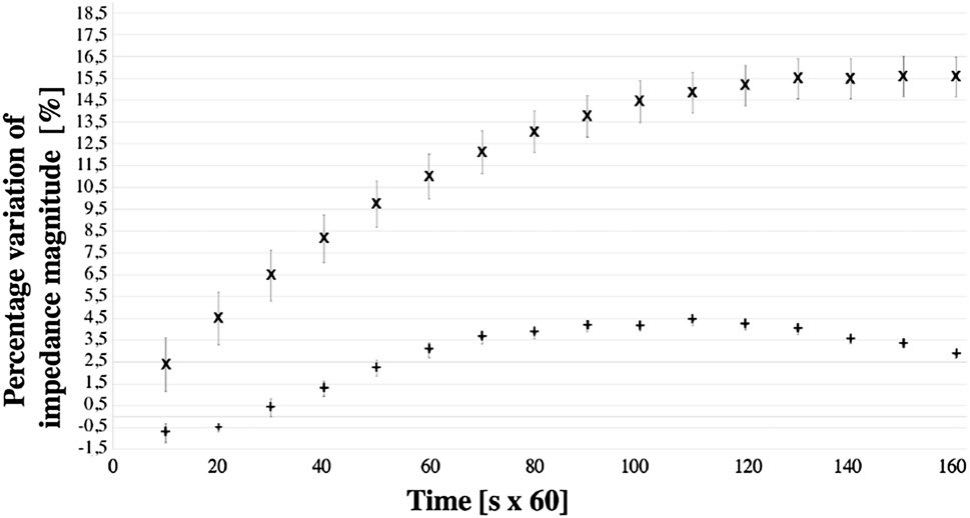
Dried eggplant drift in 2-($\times$) and 4-wire ($+$) configuration (average on 10 measurements).

#### In-vitro tests

Eggplants were selected for in-vitro tests owing to their recognized capability of emulating the electrical behavior of human skin^15,16^. In particular, *drift, sensitivity, nonlinearity, repeatability, reproducibility* and *accuracy* were assessed. For each metrological characteristic, experimental results of a *generic* and a *personalized* model, or 2 and 4-wire measurement are compared. Furthermore, for the parameters of greatest interest, the differences between the two abovementioned set-ups, are also pointed out. The generic model was identified off line on all the samples during the metrological qualification tests. The personalized model was identified on line on the specific subject tissue in the particular measurement conditions. The general model points out the performance of a system applicable to a generic individual independently from the condition of use and the tissue. The personalized model points out the performance of the proposed insulin meter with a personalized model identified in every condition of use.

#### Experimental setup

Tests were carried out on 29 peeled and dried eggplants to attenuate impedance intrinsic uncertainty arising from water evaporation during measurements (9 on a setup with inter-electrodic distance of 12 mm, and 20 on a setup with a distance of 5 mm). Drying was carried out under the same conditions: 2 h, temperature of 23 °C, and relative humidity of 50%. Each eggplant was cut as a specimen of $10 \times 4 \times 4$ cm. *12 mm setup:* The FIAB PG500 electrodes ($28 \times 36$ mm) were placed on each specimen, cut lengthwise in half (to obtain four electrodes $14 \times 36$ mm) and placed at a distance of 12 mm. Measurements were carried out by imposing a sinusoidal voltage amplitude of 20 mV, at a frequency of 1 kHz. Preliminary measurements were carried out for each sample before infiltration, in order to both leave aside the initial value and improve inter-species reproducibility. Lilly’s Humalog pen solution, containing fast-acting insulin (100 UI/ml, where each UI indicates 0.0347 mg of human insulin) was used. Insulin was injected by five consecutive steps of 0.05 ml (thus reaching a total volume of 0.25 ml), at a depth of 8 mm (PIC Insumed31G syringe with G31 $\times$ 8 mm). *5 mm Setup:* The instrument sensitivity was improved by optimizing the setup in order to enhance also as much as possible the usefulness in diagnostic-therapeutic applications. To this aim, the inter-electrode distance, the injection depth, and the amount of insulin were varied. Two electrodes FIAB PG500 ($28 \times 36$ mm) were cut lengthwise to obtain four electrodes of $7 \times 36$ mm. The inter-electrodic distance was set at 5 mm and an insulin injection was performed using a 100 U/ml Insulin Pen at a depth of 4 mm. 2 IU (i.e. 20 $\upmu$l of insulin solution) were administered for five consecutive injection, reaching a total 10 IU dosage (i.e. 100 μl of solution) in each individual experiment; this experimental setup was chosen to closely reproduce insulin injection technique and doses commonly used in diabetes care. After each injection (12 mm and 5 mm setup), impedance measurements were repeated to reduce uncertainty.

#### Drift

The four-wire configuration attenuates the impact of electrolytic gels on the measurement drift, a strong impact factor in previous studies^10^. In Fig. 4, the trends of the progressive penetration of the gel are pointed out for the two and four electrode configurations. In two-wires configuration, the gel progressive penetration into the tissue results in a decrease in contact impedance within 160 min. The four-wires configuration neutralized this effect: The drift is 2.9% (Fig. 4).

#### Sensitivity

**Figure 5:**
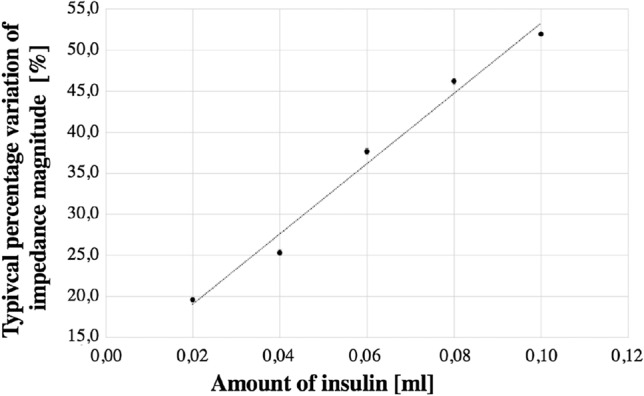
Percentage impedance magnitude variation versus amount of insulin solution in in-vitro experiments in 5 mm setup.

The insulin meter sensitivity was assessed by the slope of the linear model. The average value is equal to 24.7 ml^−1^ in 12 mm and 497.3ml^−1^ in 5 mm setup. In particular, for a variation of 1 ml of insulin solution, a corresponding variation of 497.3% is appreciated in percentage impedance with respect to the initial value before the injection. The typical trend of results in eggplant in 5 mm Fig. 5 indicates the percentage change in impedance in relation to the drug quantity. A sensitivity improvement of 20% due to the 4-wire configuration is highlighted^10^.

#### Non-linearity

The non-linearity was determined through one-way ANOVA (ANalysis of VAriance), as the standard deviation of the residuals of the linear model. The typical percentage value (expressed as the average ± 1-σ of the sample mean) for 5 mm is reported in Table 1 for personalized and the generic model. The personalized model exhibits a non-linearity always lower than the generic model.

**Table 1:** Transducer non-linearity 5 mm.

Eggplant sample	Generic %	Personalized %
# 1	9.7	4.6
# 2	5.3	5.1
# 3	4.6	4.6
# 4	5.2	1.6
# 5	9.9	9.8
# 6	1.6	1.6
# 7	3.2	1.4
# 8	4.8	3.4
# 9	6.8	4.4
# 10	6.1	5.1
# 11	3.7	2.0
# 12	3.9	2.2
# 13	4.5	3.9
# 14	5.0	4.8
# 15	2.5	2.5
# 16	4.6	3.3
# 17	2.8	2.5
# 18	11.5	9.9
# 19	4.8	2.3
# 20	9.5	6.0
Mean	$5.5 \pm 0.6$	$4.0 \pm 0.5$

#### Repeatability

**Figure 6:**
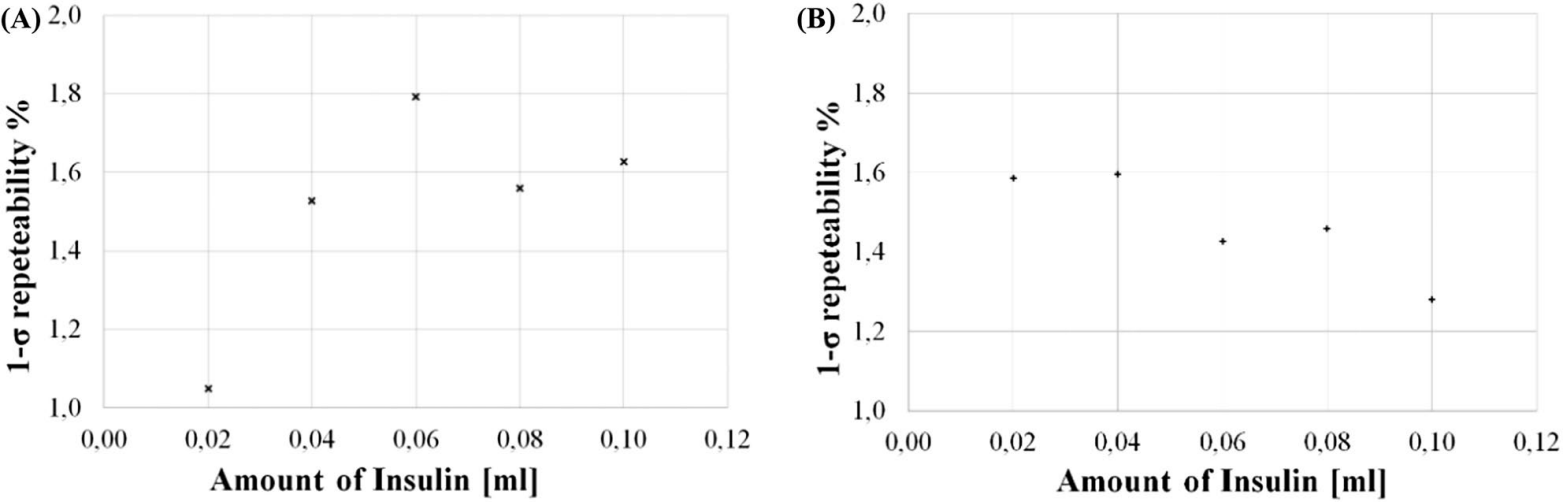
1-σ-repeatability versus amount of injected drug in in-vitro (**A**) and ex-vivo (**B**) experiments.

The 1-σ repeatability of the 12-mm and 5-mm setups was assessed as the percentage variation with respect to the initial impedance value at varying the amount of injected drug. Average percentage values of 0.31% and 1.3% were determined per the 12-mm and the 5 mm setup, respectively. The trend for the former setup is reported in Fig. 6(A).

#### Reproducibility

A 1-σinter-individual reproducibility of 0.1% and 0.7% was assessed for the personalized and the generic solution, respectively, for the 12-mm setup. The reproducibility for the optimal setup are 2.4% and 2.7% for the personalized and generic solution, respectively.

#### Accuracy

**Figure 7:**
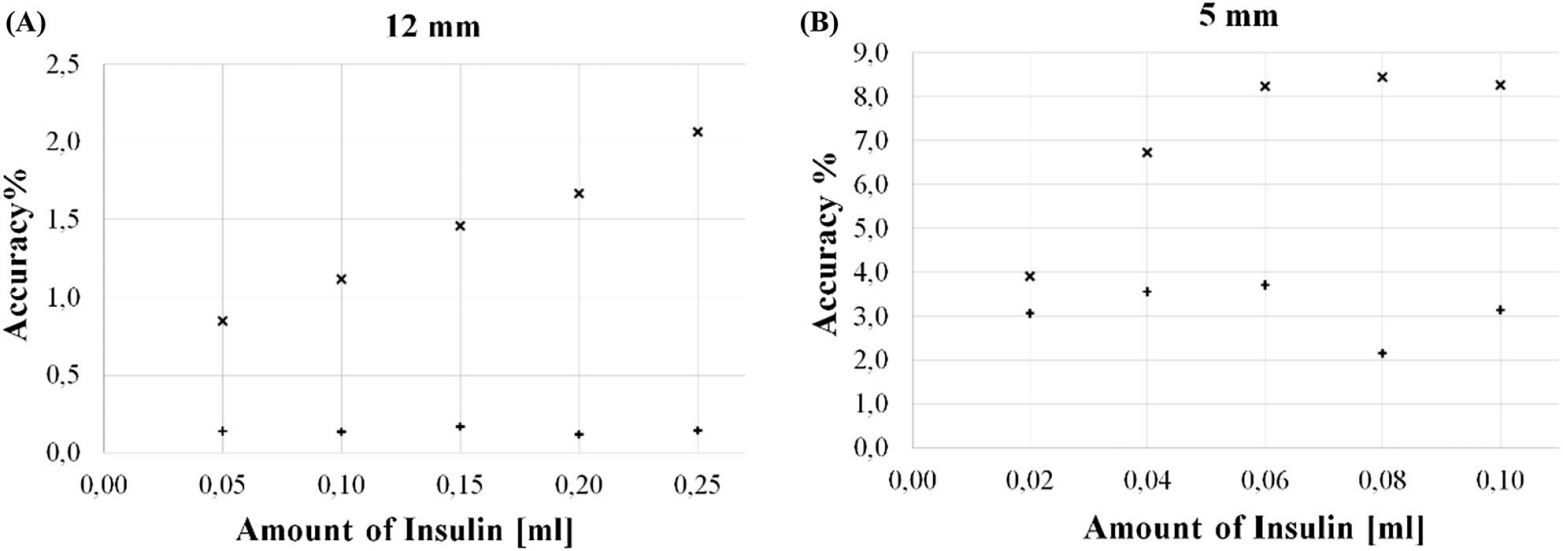
Percentage accuracy of personalized (+) and generic ($\times$) model versus amount of insulin, in in-vitro experiments for (**A**) 12-mm and (**B**) 5-mm setup.

In Fig. 7, the RMS of the deterministic error is reported at varying the injected insulin, for both the personalized (+) and the generic model ($\times$), in the (A) 12-mm and (B) 5-mm setup, respectively.

### Ex-vivo tests

**Figure 8:**
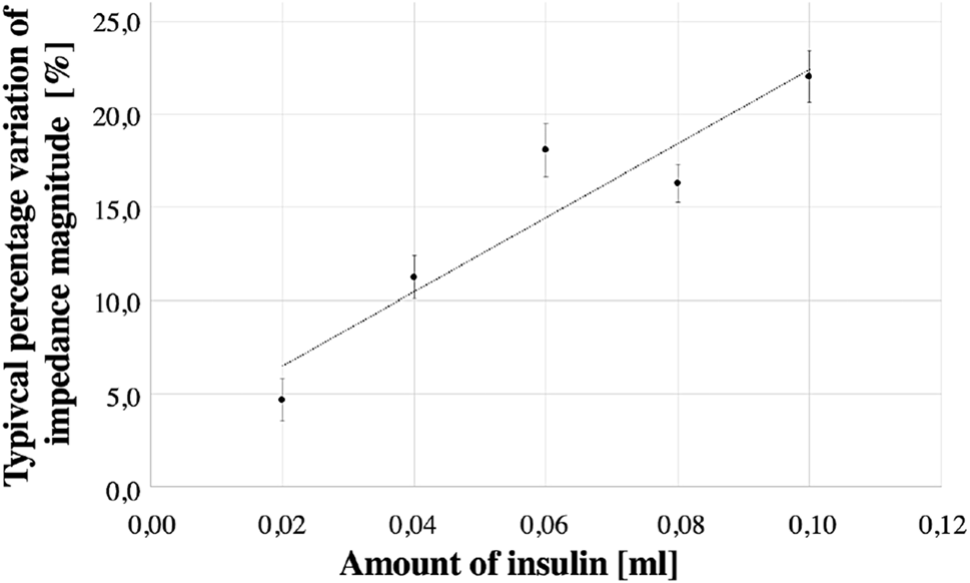
Typical percentage magnitude variation versus insulin solution in ex-vivo experiments.

According to several studies, the pig has properties very similar to human skin. The permeability of the membrane^17^, as well as the epidermal thickness and the lipid part, are indicated to be very similar to those of humans^18,19^. For this reason, ex-vivo tests were carried out on 15 samples of abdominal non-perfused muscle of different pigs, under controlled conditions ($25^{\circ }$C, and relative humidity of 50%). All methods were carried out in accordance with relevant guidelines and regulations. All the samples were provided by a local abattoir in compliance with the regulations on products of animal origin intended for human consumption. The experiments were not the cause of the pain, suffering, or death of any animal. The parts have dimensions of of $7 \times 7 \times 4$ cm, and the surface was treated before each test. The optimal setup was used, by carrying out five consecutive injections of 2 insulin units (IU), corresponding to 20 $\upmu$l of insulin solution, at a depth of 4 mm via insulin pen. The resulting metrological characteristics are represented analogously as in eggplant experiments. In Fig. 8, the trend of the percentage variation of the impedance module is reported at varying the injected insulin.

**Figure 9:**
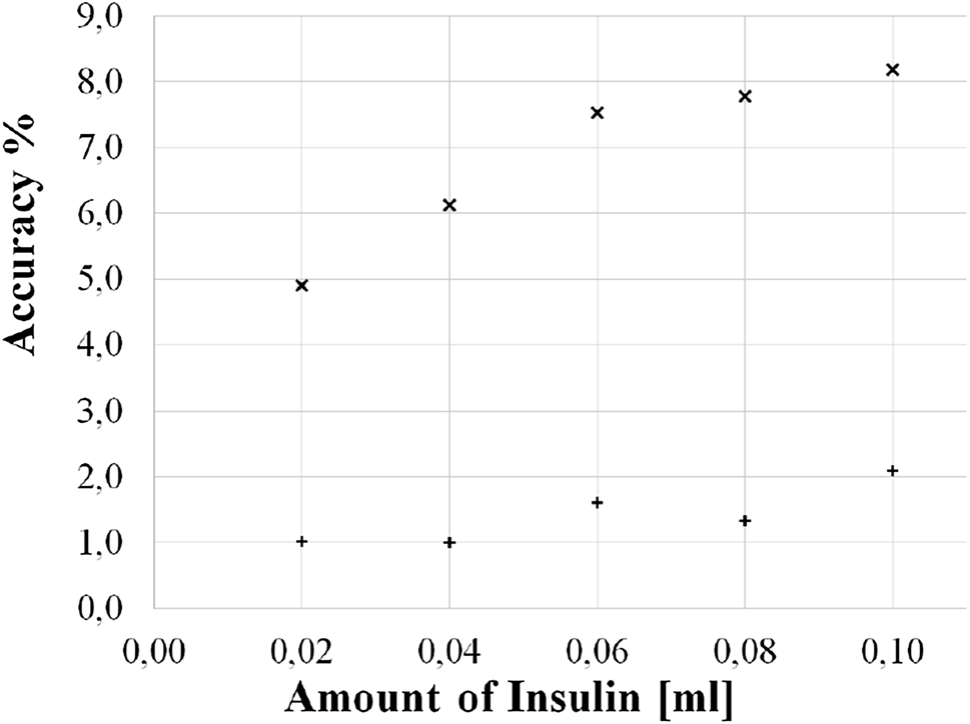
Percentage accuracy of personalized (+) and generic ($\times$) model versus amount of drug in ex-vivo experiments.

In Table 2, the sensitivity, the non-linearity, the 1-*sigma* repeatability, the reproducibility, and the accuracy measured in the pig and in the eggplant tests are reported.

**Table 2:** Metrological pig abdominal non-perfused muscle characteristics.

	Pig	Dried eggplant
Sensitivity (ml^−1^)	157.2	497.3
Non-linearity (%)	2.1	4.0
1-σ repeatability (%)	1.6	1.3
Reproducibility (%)	2.4	1.9
Accuracy (%)	Fig. 9	Fig. 7(B)

For the sake of the comparison, the same setup with inter-electrode distance 5 mm was used. In Figs. 6(B) and 9, the 1-*sigma* repeatability and percentage accuracy, respectively, are indicated for both the generic and the customized model.

### Preliminary in-vivo tests

**Figure 10:**
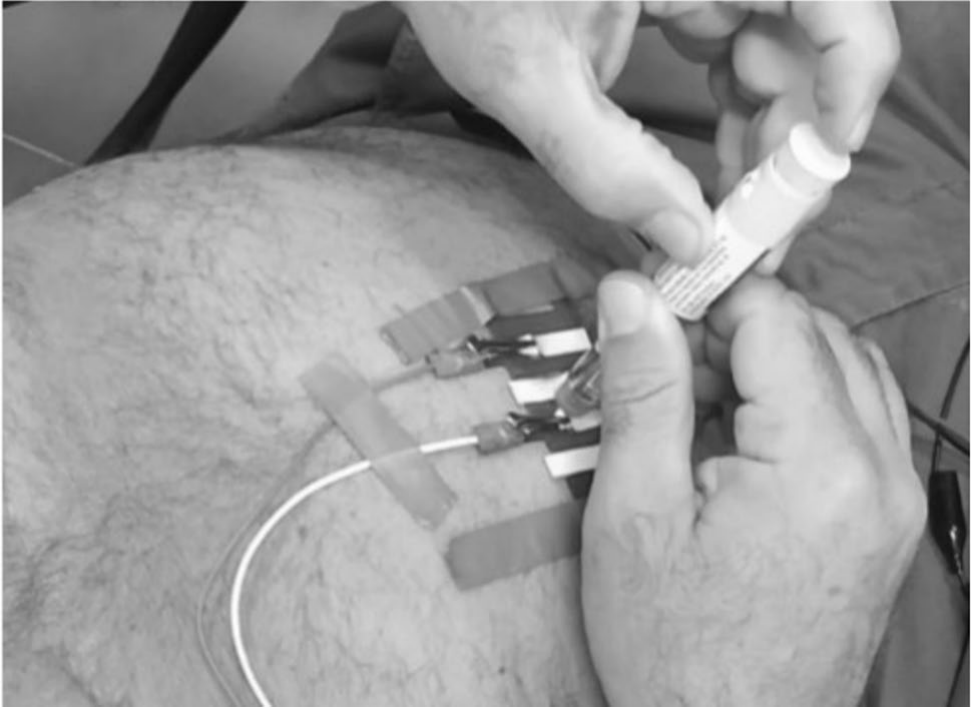
Insulin injection during in-vivo experiments.

**Figure 11:**
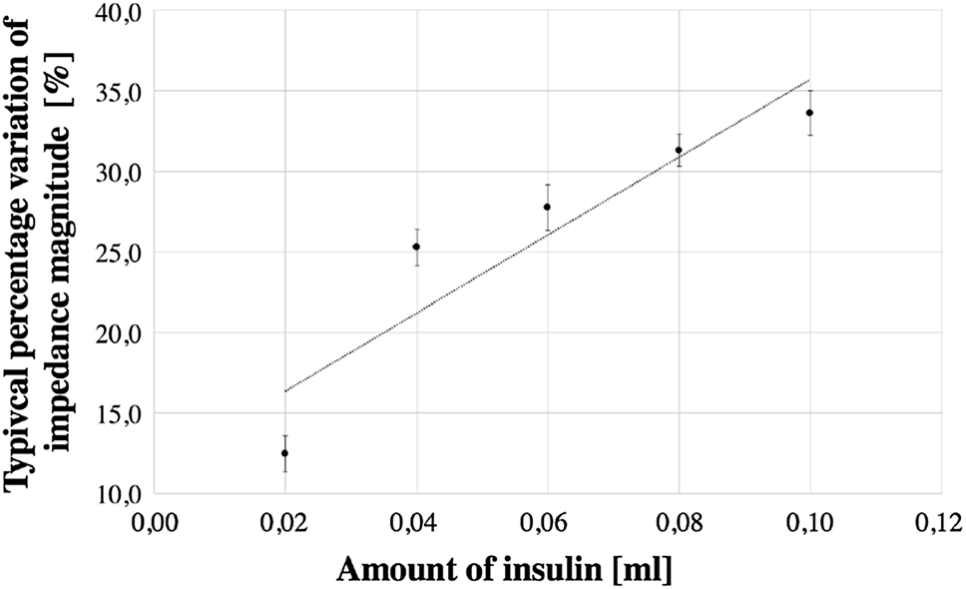
Typical percentage magnitude variation versus insulin solution in in-vivo experiment.

Some in-vivo tests were carried out in order to validate the proposed transducer on a voluntary human subject affected by type 1 diabetes. The Ethical Review Board of the University of Naples Federico II approved the research. The volunteer is a patient already undergoing diabetic therapy, with fast insulin administered as bolus in suitable amounts 20 min before meals. A commercial injectable solution of insulin Lilly’s Humalog of 100 U/ml, of 3 ml, in a configuration of pre-filled pen, was used. The experiment was carried out by measuring through the Insulin Meter the impedance just during the bolus injection of the patient. The same setup with inter-electrode distance 5 mm and injection depth 4 mm was used. Insulin was administered at successive steps of 0.02 up to the final amount of the patient therapeutic value of 0.10 ml (Fig. 10). For each administration step, the impedance was measured, according to the protocol established in in-vitro and ex-vivo tests. In Fig. 11, the typical results of the experiment are reported. A monotonous increasing trend is inferred, and an instrument sensitivity capable of appreciating the insulin variations typical of clinical practice. The linear model is proved to be useful also on human subjects, as well as optimal to describe the trend of the phenomenon already identified in in-vitro and ex-vivo tests.

After the first measurement session (Table 3, first row), the instrument was upgraded by optimizing the following parameters: (1) applied voltage, increased up to the limits allowed by the safety thresholds, and (2) impedance of the cables connection and contacts.

Then, further tests were carried out on the same subject over 2 months, up to a total of 300 measurements (namely, 50 for each daily session). 1-sigma repeatability was decreased by an order of magnitude on average with respect to the first session of tests (Table 3, last column). Among other advantages, the increase in repeatability allowed to double the resolution of the steps of the measured quantity (from 2 to 1 IU per step). The experiments confirmed the well-known problem of reproducibility for bio-impedance measurements^20^, also at intra-individual level, mainly due to changes in the skin moisture^21^. During the six measurement sessions, the subject exhibited impedance values with an uncertainty band of 42 $$\Omega $$ compared to an average of 157 $$\Omega $$. This variability in the initial impedance does not arise from the different frequency values of the stimulus signal. Even with the same applied frequency, the differences between the test sessions are significant. In the different measurement sessions, also sensitivity exhibits poor reproducibility: average of 98 ml$$^{- 1} $$ and uncertainty band of 80 ml$$^{- 1} $$.

The proposed method proved to be relevant in managing the impact of inter- and intra–individual reproducibility. The method allows the insulin emergence model to be built at each administration. Thus, the uncertainty is reduced to the sole contribution of the measurement non-repeatability. The optimized version of the proposed micro-transducer guarantees an average percentage of 1-$$ \ sigma $$ repeatability lower than 0.5%. Even considering the worst-case sensitivity of 47 ml$$^{- 1} $$, if a bolus is administered (typically with a value of 10 IU), a significant variation of to 4.7% of the initial impedance, or 10 times greater than the 1-sigma repeatability, can be appreciated.

**Table 3:** In vivo experimental results.

Dosage	Frequency	Initial impedance	Sensitivity	Non linearity	1-σ repeatability
[IU * nr. step]	(kHz)	Value ($\Omega$)	(ml$^{-1}$)	(%)	(%)
2 IU * 5	1	343	242	16	4.17
1 IU * 6	1	220	$-$ 113	12.1	0.72
	10	179	408	3.8	0.39
	10	56	$-$ 101	6.5	0.41
	50	78	197	5.5	0.41
	50	66	$-$ 47	13.5	0.31

## Methods

In-vivo experimental protocols were approved by the ethical committee of the University Federico II. All methods were carried out in accordance with relevant guidelines and regulations.

### Laboratory tests

Laboratory tests were carried out trough an equivalent circuit obtained as a parallel between a capacitor [0.5 nF] and a resistor [42–2500 $\Omega$] (realized by 1433-M Decade Resistor and a Standard Capacitor Type 509-F both of General Radio). 32 measurements were performed at the frequencies of [100, 120, 10,000, 10,000, 20,000] Hz, with a sine wave amplitude of 100 mV, at a temperature of 23 °C and humidity of 50%. The impedance module of the RC loop were assessed as average and standard deviation, and compared with LCR Meter Agilent 4263B from Agilent Technologies as a reference.

### In-vitro tests

The in-vitro tests were implemented on peeled eggplants (size $10 \times 4 \times 4$ cm) dried under the same conditions: 2 h, temperature of 23 °C, and relative humidity of 50%. Two different inter-electrode distance set-ups were used: 12 mm and 5 mm. For the (1) 12 mm inter-electrode distance were used two FIAB PG500 electrodes ($28 \times 36$ mm) cut in half, using a sinusoidal voltage amplitude of 20 mV, at a frequency of 1 kHz. Lilly’s Humalog pen solution, containing fast-acting insulin (100 UI/ml, where each UI indicates 0.0347 mg of human insulin) was used. Insulin was injected by five consecutive steps of 0.05 ml (thus reaching a total volume of 0.25 ml), at a depth of 8 mm (PIC Insumed 31G syringe with G31 $\times$ 8 mm). For (2) 5 mm inter-electrodic distance were used two FIAB PG500 electrodes, cut lengthwise to obtain four electrodes of dimensions 7 $\times$ 36 mm. The insulin injection was performed using at 100 U/ml Insulin Pen at a depth of 4 mm. 2 IU were administered for five consecutive injections (reaching a total volume of 100 $\upmu$l in each individual experiment); this experimental setup was chosen to closely reproduce insulin injection technique and doses commonly used in diabetes care. . In both cases (12 mm and 5 mm setup), preliminary measurements were carried out for each sample before infiltration.

### Ex-vivo tests

The ex-vivo tests were performed on abdominal non-perfused muscle of different pigs, under controlled conditions 25 °C, and relative humidity of 50%. All methods were carried out in accordance with relevant guidelines and regulations. All the samples were provided by a local abattoir in compliance with the regulations on products of animal origin intended for human consumption. The experiments were not the cause of the pain, suffering, or death of any animal. The parts have dimensions of $7 \times 7 \times 4$ cm. The surface was treated before each test: the physiological residual liquid was removed with a buffering gauze and a disinfectant wipe was used to remove residual germs and bacteria on the surface. The optimal setup (5 mm inter-electrodic distance) was used at a depth of 4 mm via Lilly’s Humalog insulin pen by carrying out five consecutive injections of 2 IU (reaching a total volume of 100 μl). FIAB PG500 electrodes sized at $7 \times 36$ mm with a sinusoidal voltage amplitude of 20 mV at a frequency of 1 kHz were used.

### Preliminary in-vivo test

The study was conducted in accordance with the Declaration of Helsinki. The experimental protocol was approved by the ethical committee of the University Federico II. Written informed consent was obtained prior to the experiment. The volunteer is a patient already undergoing diabetic therapy, with fast insulin administered as bolus in suitable amounts 20 min before meals. The same setup with inter-electrode distance 5 mm and injection depth 4 mm was used. In the first measurement session the insulin was administered with a Lilly’s Humalog pen at successive steps of 2 U up to the final amount of the patient therapeutic value of 10 IU (equivalent to 100 $\upmu$l). The parameters of the Insulin Meter system sinusoidal voltage amplitude of 20 mV and a frequency of 1 kHz were set. In subsequent sessions, insulin was administered in six successive 1 IU steps and signal frequency was varied in the range $\{$1 kHz, 10 kHz, 50 kHz$\}$.

## Conclusion

A transducer was designed to monitor insulin levels in clinical diabetology practice. The micro-instrument shows in in-vitro experiments a sensitivity of 497.3 ml^-1^, a non-linearity of 4.0%, a 1-σ repeatabilily mean of 1.3% and a reproducibility of 1.9%. In ex-vivo experiments shows a sensitivity of 157.2 ml^-1^, a non-linearity of 2.1%, a 1-σ repeatbility mean of 1.6% and a reproducibility of 2.4%. These values are suitable for clinical applications. In clinical practice, the insulin meter allows to measure the variation of units of insulin, relating it to the slope of the linear model, by confirming the preliminary data identified in^14^. Commercial “insulin pumps” analyze the amount of glucose in the blood in order to identify the insulin absorption curve. In this way, suitable software packages (e.g., “Bolus Wizard”^22^) determine the insulin to be administered during the day.

Main drawback is that the amount of glucose in the blood is not a direct indication of the punctual absorption of the insulin. Therefore, the possibility of monitoring the disappearance of insulin from the injection site would make the bolus wizard a fundamental tool for PWD. Furthermore, the possibility to estimate the insulin disappearance curve provides a sound basis for the analysis of personalized therapies in different conditions of use.

Systematic clinical studies will be carried out on PWD to validate the results on eggplants and pig abdominal non-perfused muscle. These studies shall lead to a bolus wizard with insulin administration personalized for each individual.
